# Roadblocks in education amidst global crisis—A study based in India

**DOI:** 10.1371/journal.pone.0292465

**Published:** 2023-10-17

**Authors:** Surbhi Dayal, Pratibha

**Affiliations:** 1 Humanities and Social Sciences, Indian Institute of Management Indore, Indore, Madhya Pradesh, India; 2 Learning & OD, Kalpataru International Projects Ltd., Mumbai, Maharashtra, India; Fiji National University, FIJI

## Abstract

**Background:**

The outbreak of the COVID-19 pandemic compelled the closure of educational institutions and forced students to complete nearly two years of schooling online, impacting their physical and emotional development tremendously. This exploratory study investigates the wide-ranging impact of online education on Indian students during the COVID-19 pandemic and discusses the challenges exacerbated by disparities in access to digital devices and reliable internet service. The paper also focuses on the physical and mental health issues that arose in student cohorts as a result of the abrupt shift to online learning, and investigates the relationship between students’ socioeconomic status and the nature and frequency of health issues experienced by them.

**Methods:**

A total of 832 respondents completed a 40-item survey that was administered online and through interviews. The paper analysed the impact of access to digital resources and teachers’ training in information and communication technology on the perception of the quality of education provided by the institutions. We further analysed the impact of the adoption of online educational platforms on students’ mental and physical health.

**Results:**

The study found a positive relationship between the number of hours spent online, and the physical and mental health issues experienced by students. Participants reported an overall higher perception of stress and anxiety, loss of concentration, and dissatisfaction with the quality of education. Our data suggest that COVID-19 has exacerbated the digital divide.

**Discussion:**

Urgent investments are needed to provide universal access to reliable internet services, and to develop a pedagogy that supports an agile and adaptable educational system, capable of providing effective learning and evaluation, while supporting students’ physical and mental health.

## Introduction

The World Health Organization declared COVID-19 a pandemic on March 11, 2020 [1]. Governments throughout the world implemented a total closure of all operations, with the exception of emergency and vital services, and advised people to stay inside their homes in order to stop the spread of the coronavirus. On March 23, 2020, the Indian government imposed a nationwide lockdown, which resulted in educational institutions shutting down [2]. During the lockdown, over 80% of government school students in Odisha, Bihar, Jharkhand, Chhattisgarh, and Uttar Pradesh were not given any instructional resources to study from home [3]. According to UNESCO [4], approximately 1.6 billion students were impacted in 194 countries, accounting for nearly 90% of all enrolled learners. India had the second-longest school closure duration during COVID-19 with 82 weeks, just behind Uganda, which had 83 weeks of school closures due to the pandemic. Educational institutions, whether governmental or private, were compelled to resort to online and remote learning.

The spread of COVID-19 disrupted the world’s traditional modes of education and introduced new, unforeseen challenges. To continue to impart education to students, institutions shifted from physical classes to an online and remote mode of instruction. Due to the abrupt transition from the old pedagogical paradigm to online learning, students and educators were expected to become acquainted quickly with the digital tools that educational institutions employed. However, the quality of the education delivered suffered, due to a lack of training for all stakeholders involved. The availability of technological tools and internet access, a prerequisite for online learning and a solution for remote teaching, also became a key obstacle towards achieving it [5].

Global studies on the impact of the COVID-19 pandemic and the subsequent suspension of educational institutions have examined a variety of issues affecting students in both schools and universities [6]. An overall deterioration in mental health has been observed across all student groups, with varied levels of psychological concerns manifesting as a result of students dealing with unexpected circumstances [7]. The impact has been extensive for students who have had to deal with online learning without proper financial and familial support, as well as infrastructure deficits, particularly in relatively underdeveloped areas. It is worth noting that the pandemic brought to the fore problems at the policy and institutional levels within the greater socio-economic framework [8]. Existing disparities in access to opportunities and education among students have been worsened, and gaps and cracks in the education sector exacerbated. Thus, there has been a concomitant increase in the number of recent Indian studies analyzing the present state of the education sector, which, while proposing novel strategies for how schools should prepare to open up, anticipate the need for further digitalization and revamping of the methods used to disseminate education in India [9].

Due to the unpreparedness of the education system, several countries, including India, have had to formulate ways of disseminating education without much planning, which has led to the use of non-standardized methods of taking classes and a waning in students’ engagement and attention. Leveraging the potential and effectiveness of online learning and the need to strategize to bolster novel ways of educating students has assumed utmost importance. The relevance of analyzing students’ perceptions, attitudes, and current performance, is critical for policymakers to take into account to establish useful, effective programs in the education sector [10]. The long-term effects of the pandemic, as well as the concerns it has raised regarding the possibility of imparting education through innovative means and methods, require careful examination to make effective provisions in the post-pandemic period [11]. The transformation of the education sector in the post-pandemic period needs to be cognizant of technological bolstering, digital ramp up, institutionally mandated student support, reconsideration of assessment methods, and a plan of action to bring back those students who were left out of the fold of education during COVID-19 [12].

### Internet and communication deficit

The smooth functioning of digital classrooms depends on reliable internet access. Even though 93% of the world’s population lives in areas with internet access, only 53% of them have the means to utilize it [13]. This is reckoned to be a significant barrier in the educational process as the educational experience is hindered due to internet connection unreliability or overloaded servers. The communication lag obstructs the teaching’s natural flow, which lowers students’ participation within the class and decreases their interaction with teachers. To overcome the limited interaction due to digital modes of education, many educators used interactive tools to keep students engaged, but with mixed success [14]. Despite all efforts, a large number of students were not able to partake productively in learning due to the lack of digital resources.

According to a survey conducted by the International Association of Universities (IAU), almost 91% of higher education institutions have a system in place to disseminate education to their students; but are challenged with maintaining clear and effective communication channels with staff and pupils. Nearly 59% reported that they had to cease operations in a survey conducted between 25^th^ March 2020 and 17^th^ April 2020. Africa had the highest number of educational institutions (77%) remaining closed for online/offline mode of functioning [15].

The internet is becoming more popular, but many people in developing countries, particularly in Southeast Asia, do not have access to it. Lack of digital devices hinders learning, especially for low-income and rural students [16]. In Southeast Asia, three countries dominate internet usage: Singapore, Brunei, and Malaysia. It is well known that not every student can afford a digital device [17]. Many students do not have constant access to digital devices, and family members share devices, creating a digital divide [18].

Students in rural and remote areas struggle with internet access, limiting their educational opportunities. Factors related to students’ home environments, and a lack of proper digital infrastructure or training result in school children in rural areas lagging behind in adopting new e-learning practices, developing mobile expertise, and being able to timely submit assignments. The meager availability of basic funds, poor communication, and lack of adequate guidance for students has been recognized to contribute to educational shortfalls in India’s North Eastern states [19]. While some educational institutions also provided recorded classroom sessions for students to review, limited internet data made it difficult to download the entire lecture. To overcome this obstacle, voice-over PowerPoint presentations which require less internet bandwidth to download, were used [20]. All this suggests that online learning and teaching have increased the burden of complete transition into external applications, communication overload, and stress for both teachers and students, leading to adverse impacts on the latter’s academic performance and mental health.

Additionally, the gendered interplay of COVID-19’s negative impact on the education sector is critical to assess. Insufficient access to electronic gadgets, inadequate parental involvement, or an inconducive home environment for education were significant barriers for both genders, with girls slightly more affected. The burden of domestic chores, the priority of devices to male students, and the issue of having shared phones to study amongst multiple siblings, were all observed. This was corroborated by Delhi government statistics, which showed that only 60% of students participated in remote learning in the initial stages of the lockdown, most likely due to the reverse migration crisis. In this analysis, the number of girls who suffered a language barrier with English study material was significantly higher (17%) than boys (4%) [21].

### Quality of education dissemination

School plays a significant role in promoting socio-emotional development, and contributes to the overall well-being of students. Schools serve as a space for a child’s overall development and a hub for communication and learning. A classroom, or physical learning environment, have been shown to improve students’ emotional and behavioral well-being. Attending school is beneficial because group activities are considered therapeutic. Education also instills hope for the future in children and can absorb the energies of adolescents and young people [22]. Students who attend class in-person additionally benefit from positive relationships with educators and access to institutional resources.

Physical classes provide personal interaction between the teacher and pupil, but in online classes, educators can find it difficult to gauge the degree to which students comprehend the subject matter. If technical issues arise, online students may be unable to interact, submit assignments, or access study materials. This causes frustration, poor performance, and disinterest in studies [23]. Additionally, the effects of school closures, social segregation, and isolation from the outside world can expose pupils to malnutrition, and make them potential witnesses and victims of domestic violence, while also increasing their stress and anxiety levels [24].

Assessing students’ progress is considered an essential part of the educational process. Teachers, while concerned about academic dishonesty in online assessments, struggled to prevent unethical student behaviors. Due to a lack of specific ICT solutions, educators had to reduce exam time and design more complex questions [14]. In our study too, it was found that at times, internet fluctuation became a major issue during online exams. Therefore, accurate and adequate assessment of students also became one of the major challenges for teachers.

### Physical and mental health-related issues

The closure of schools has diminished the level of confidence in students, which is essential to maintain in challenging times [25]. This is seen as one of the crucial concerns that students are now facing, among many other social, physical, and emotional stresses.

Student health is one of the most important concerns arising from online learning. No natural disaster has affected mental health like the COVID-19 pandemic. Education disruptions can cause significant stress to students, affecting their academic performance and mental well-being [26]. Students’ decreased motivation and fluctuating emotions hampered the effectiveness of online learning, and the pandemic increased mental stress due to the sudden shift to online education and increased screen time, which, it has been suggested, can in turn manifest in the form of disturbed and irregular sleep patterns [27]. Among Indian remote learning students, it was found that 70.7% agreed that elevated screen time caused by online classes and extra social media use affected their overall devotion of time to other activities for leisure and contributed to a reduction in physical activity. Poor sleep patterns and eating habits were reported by 52% respondents [28]. Online education and testing also caused health issues like eye strain and headaches in students [29], as well as raised anxiety levels, with 82.7% reporting stress due to online classes [18].

Remote learning, situational issues, and self-management instability can have a grave impact on an individual’s learning. Factors such as having trouble accepting e-learning is positively related to the risk of experiencing stressful situations. Due to additional social circumstantial stress from life events, females displayed a higher stress level than males [30]. Pandemic-driven remote learning saw a greater increase in issues for students with disabilities or previous co-morbidities and health concerns. School administrations making sudden changes in online class facilitation methods and institutional policies or requirements, have reported putting additional stressors on such students. For students from the economically weaker sections, along with fiscal and familial issues, greater levels of severe discrimination, difficulty concentrating, sleeping, and having negative feelings, as well as the overall struggles faced as a result of prolonged isolation, were also noted to be substantial [31]. Even if students from low-income families were intrinsically motivated, their performance and drive to work online, suffered as a result of external disruptions [32]. Studies predict that ICT-related obstructions coupled with the fear of missing an educational year due to the pandemic can manifest in an increase in psychological problems [33].

In the above context, we studied the adaptation process to online learning and the various challenges faced by students. A key question that was explored over the course of the study was whether students were satisfied with the quality of online education and the assessment system. The study discusses the challenges that were aggravated by the inequalities in access to digital devices and reliable internet service. Further, it examines the physical and mental health problems that surfaced in student cohorts due to the sudden shift to online learning and the difficulties of adjusting to the new normal, as well as the relationship between various health challenges faced by students and their socio-economic status. Based on these explorations, recommendations are proposed in this paper to help policymakers and governments cover deficits and bridge gaps created in the education sector during the pandemic.

## Method

This was an exploratory study, and a combination of online surveys and interviews were used for data collection. We developed a detailed survey using Google forms which included 40 questions related to different aspects of online learning: school-life balance, and the impact of online learning on the physical and mental well-being of students. A pilot survey was circulated to 50 students studying in all three types of educational institutions in India: high schools, coaching centers, and premier higher education institutions. Based on their responses, it was modified and was subsequently circulated via various online platforms like email, and WhatsApp to the targeted population. After circulating the survey form, we followed up over the phone or messages with the respondents to ensure that they fit the desired demographic and were completing the form. Apart from these surveys, 130 online interviews were conducted to get more in-depth information from the respondents. The Research Advisory Committee on Codes of Ethics for Research of Aggrawal College, Ballabhgarh, Haryana, reviewed and approved this study. A statement was included in the survey form to obtain the participants’ written consent. In the case of minors, parental consent was obtained. The confidentiality of the respondents was maintained throughout the study.

Data were collected from June to December 2021. We recruited a targeted population of 832 students from the states of Rajasthan, Madhya Pradesh, and New Delhi. Among these, 52% students were studying at premier educational institutions like the Indian Institutes of Technology, Indian Institutes of Management, and top Central universities, and the rest were in high school, and/or studying at coaching institutes, preparing for professional exams. The population of male respondents was 62%, and the rest (38%) were females. While 48% of the respondents were 18 years of age or older, the rest (52%) were younger than 18 years of age.

The survey focused on how COVID-19 affected students’ lives, in particular how a shift from conventional to digital learning platforms had affected students’ health. It included several questions that concentrated on the experiences students underwent as a result of the transition from traditional classrooms to a digital mode of learning, and the learning gap that was caused by this shift.

## Results & discussion

Our study found that twenty-four hours of confinement at home, the physical unavailability of peers and teachers, the limited or non-availability of digital devices, electricity, and the internet, have all deeply affected students’ learning. Various issues experienced by the study participants are detailed below.

### Students’ perception if online education was fulfilling the purpose of education

The transition from traditional to online learning gave rise to several questions about the effectiveness and purpose of the education being imparted. Among the total participants, 82.5% from premier institutions and 73.6% from other institutions felt online education was not fulfilling the purpose of education, or was doing so only to a small extent. Only 22.1% of the total students agreed that online education was fulfilling its purpose. Students still preferred traditional education over online learning, as offline learning was seen as more interactive and comprehensive.

In alignment with this, in a study conducted at the onset of the pandemic in West Bengal, only 11.6% of students reported having completed more than half of their undergraduate and postgraduate syllabus. Furthermore, nearly two-thirds of students did not utilize e-pathshala at all for learning resources. This is indicative of the lack of percolation of government schemes implemented to benefit the student population in India [34].

#### Assessments

Assessments are an essential component of education that help determine if the goal of education has been fulfilled. During the COVID-19 pandemic, exams and other traditional assessment methods were suspended globally. The shift to online learning had a significant impact on assessment. As classes were online, the assessments were also shifted online, and this created difficulties for both educators and students. We asked the respondents about their satisfaction with the online assessment.

#### Satisfaction with online assessment

Only 30.6% of students from other institutions and 13.7% from premier institutions were satisfied with the online assessment during COVID-19. Compared to 69.4% of students from other institutes, 86.3% of students at premier institutions opposed online assessment. Assessments during COVID-19 were considered complex by the students. They reported cases of cheating, the disadvantages of an unreliable internet connection, and how the family helped some of the students score better marks than others. Students also reported that, at times, the exam paper set by the teacher was not appropriate for online learning. One of the respondents reported that it was heartbreaking to see that students who scored the lowest marks were getting the best grades as their families helped them cheat. Students cheat on online exams due to parental pressure, fear of failing, and other factors. A previous study found that when students are occasionally desperate to improve their grades, e-learning helps them cheat and secure better results [35]. Pressure to perform better and technical issues cause anxiety and stress in students [36]. In a study by Chakraborty et al., (2021) [18], students preferred weekly tests to get acquainted with the online assessment system and to improve their comfort level in taking online exams.

#### Exposure to online learning before COVID-19

Only 16.7% respondents had any prior experience with online learning before COVID-19. Students from premier institutions (21.2%) had better exposure to online learning than students from other institutions (12.5%). The instability of infrastructure and the unavailability of digital devices were more pronounced during the pandemic. The institutions also lacked the proper technological infrastructure to implement an online pedagogy. According to a study performed in Uttar Pradesh, while it was determined that 50% of pupils were either unable to attend classes wholly or had extremely low attendance, the lack of adequate monitoring bandwidth made it difficult for educational institutions to even determine the exact degree of truancy, let alone redress it [37].

#### Willingness to use online learning after COVID-19

In response to whether respondents would be willing to continue the online mode of learning post-COVID-19, 46.4% declined, citing the lack of in-person interactions, classroom discussion, and in-depth learning as a few of the reasons. Respondents also mentioned that they had learned everything in pen-and-paper mode and that the sudden transition into online mode had degraded their learning practice and experience. Approximately 40% percent of the respondents were unsure of the mode of learning they would prefer in the future. Only 14.3% of them were in total favor of continuing with the online mode of learning, post-COVID-19. An econometric analysis corroborates the aforementioned findings, with 48.1% students reporting discomfort and 47.3% reporting a dearth of communication between teachers in virtual classrooms. A total of 44.9% students were disinclined to study, and 30.4% could not grasp the education delivered via distance learning [38].

#### Satisfaction with online teaching and learning

While any pedagogy aims to impart content and build confidence among the students, the survey asked the respondents’ specifically about their satisfaction with online learning during COVID-19. Around 75% students expressed their dissatisfaction, while the rest were satisfied with online learning.

#### Time spent on online learning

Approximately 51% of respondents spent between four and six hours online, while 41.3% spent more than 6 hours online daily. COVID-19 appears to have created a divide between the students of premier institutions and those of other institutions, with only 29% of the latter spending more than six hours on online education. By contrast, the share of respondents at premier institutions who spent more than six hours online, stood at about 54.6%. This difference of 26% could be attributed to the additional tasks and assignments outside of regular lectures, and the shared pedagogy methods and devices at home, mainly the academic and extracurricular engagements and student tasks that were completed post lectures, in the form of group projects, discussions, and other such activities. Only 1% of respondents from premier institutions spent less than three hours online, while 14% of respondents from other institutions reported spending less than three hours.

The Indian Central Government, in 2020, advised a maximum of 30-minute sessions for pre-primary school students. Grades 1–8 were recommended to attend just two classes of 30–45 minutes each, while Classes 9–12 were directed to attend no more than four classes online of 30–45 minutes each [39].

Of the respondents from premier institutions, 65% reported having six working days, while 29% reported having seven. On the other hand, only 34.5% students from other institutions spent six or seven working days in an online environment. Additionally, it was observed that students from premier institutions worked more days or had engagements that spanned weekends. Furthermore, they reported that their class schedules remained unchanged and that they were learning more compared to their pre-pandemic classes. They occasionally sought additional appointments with teachers to clarify their doubts, or spent additional time listening to recorded lectures.

Respondents were also asked whether they found remote learning hectic. Around 75% of them from premier institutions, and 52% from other institutions, found remote learning hectic. Moreover, students mentioned that increased screen time precipitated health-related issues such as back pain, headaches, irritation, and decreased concentration, compared to the regular offline learning environment.

**Fig 1:**
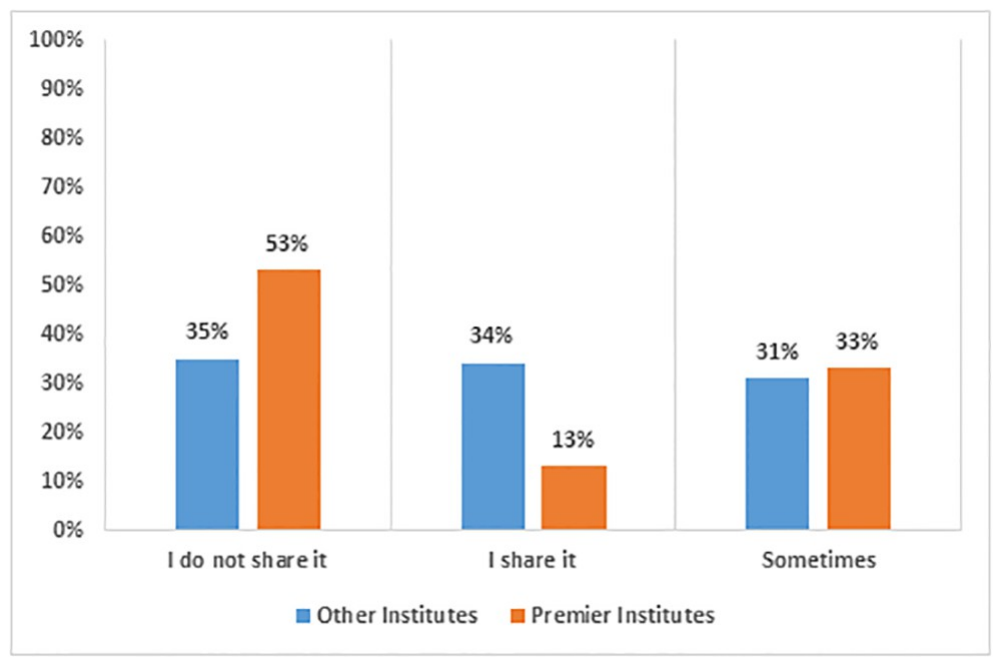
Response to device sharing.

Fig 1 shows that the availability of digital devices remained crucial during the pandemic. From other institutions, 65% of respondents mentioned that they regularly shared their digital devices with their family members to aid online learning or work from home. Respondents from premier institutions were in a better situation, as only 46% of them had to share it with other family members. This reveals a stark contrast in the access to devices according to the status of the institute. It shows that there is inequality at every level, which became evident during the COVID-19 pandemic.

#### Internet connectivity

As internet connectivity has been a pillar for online education, students were also asked about their experience of it. Internet connectivity differed drastically with city geography and the internet carrier; overall 50% among all the respondents mentioned having excellent internet connectivity while the remaining 50% had good or poor connectivity. The percentage of respondents from premier institutions, as well as other institutions, facing poor connectivity issues, was similar—34% and 32% respectively. Participants from Delhi reported better internet connectivity compared to the states of Rajasthan and Madhya Pradesh.

Disparity in access to a reliable internet connection as well as a smart device also translated into differential student engagement in other practical exercises offered by institutes. To mitigate to some extent students’ lack of practical exposure through field trips, team-building exercises, and various other activities, online education has been supplemented by webinars and workshops. Approximately 72% of surveyed students stated that they attended online webinars, but only 62% found these webinars beneficial. The remaining 28% of students did not participate in any webinars. This can also be attributed to webinars not being seen as core academic activities, thus lacking seriousness. This reflects students’ perceptions of webinars’ lacking utility in their ongoing education. Students from premier institutions attended 30% more webinars than others. From premier institutions, 86% students attended webinars because they sought to strengthen their resumes with extra activities and because they had greater access to resources.

There was overall a lack of seriousness towards online education as students were indifferent to adjusting online. They saw this as a temporary shift in the mode of information dissemination. It might well be deduced from this observation that online education lacked impact and effectiveness, and its perceived importance as a potential replacement for offline learning was substantially low.

Our observation is consistent with a study which found that 52.2% of students believed that digital sites are easy to navigate, 68% could easily access the necessary information, and 50% felt that internet learning was easy. This perceived advantage is only apparent when the sample demographic is relatively well-off and has basic digital access [38].

## Physical and mental health-related issues

With the onset of the online learning environment, there has been an increase in discussions about mental and physical health. As a result, students were also asked if they had any physical concerns due to the amount of time they spent learning online.

### Physical health issues faced by students

**Fig 2:**
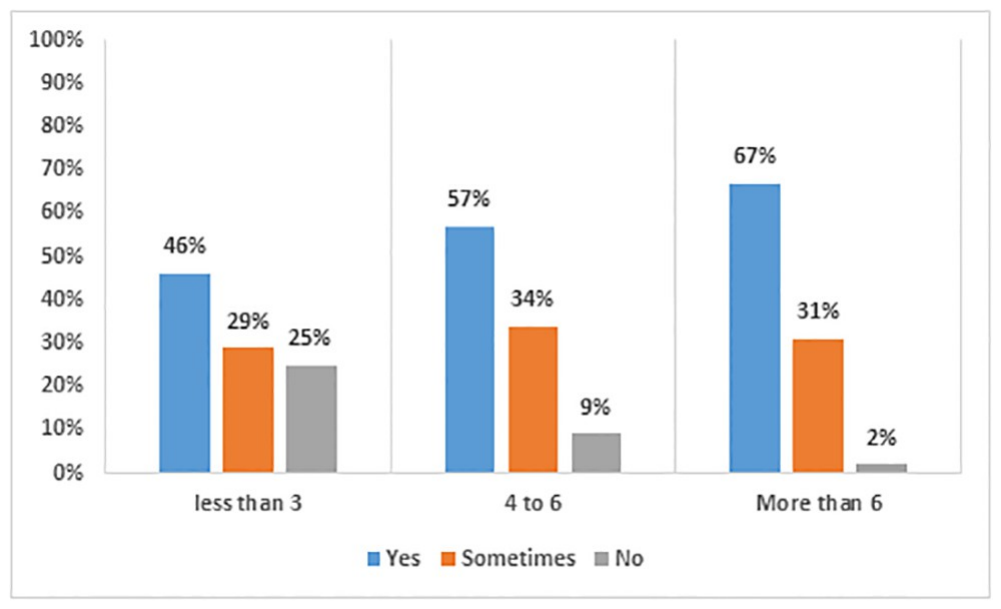
Number of working hours and physical issues faced.

Fig 2 shows that there is a positive relationship between working hours and physical health issues. Of all respondents, 75% of those who worked up to three hours had some type of physical problem. This percentage grew dramatically as the number of hours spent online increased, with 91% respondents experiencing difficulties due to learning online for four to six hours, and almost all (98%), when learning online for more than 6 hours.

**Fig 3:**
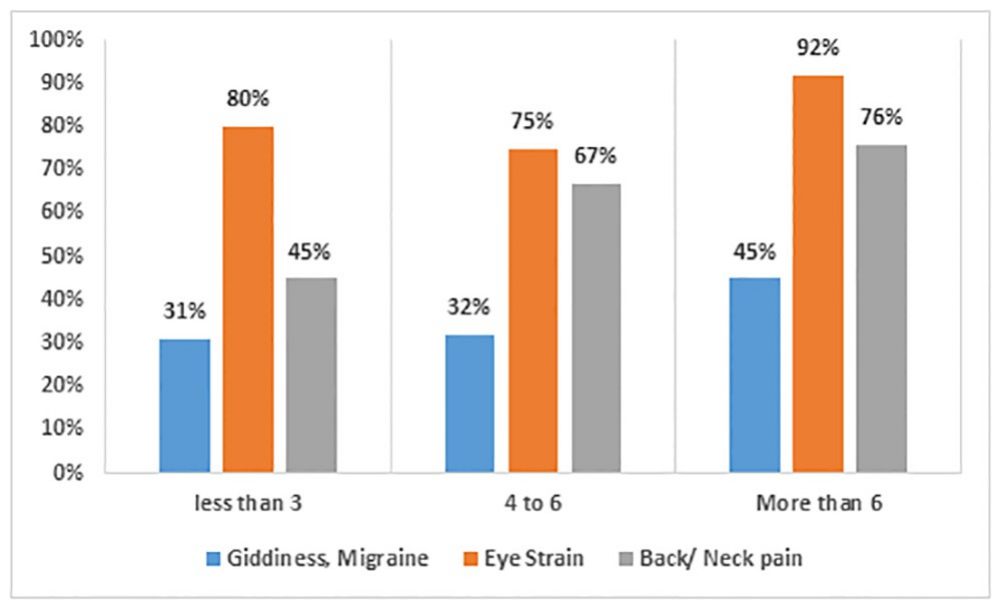
Number of working hours and type of physical health issues.

Fig 3 shows the health issues students faced due to online learning. The findings indicate that a significant proportion of the participants (92%) who worked more than 6 hours, experienced eye strain due to continuous and increased hours of screen time, possibly resulting from less-than-usual blinking while working on a device. Additionally, approximately 76% reported experiencing discomfort in their back or neck, while roughly 45% reported experiencing vertigo or migraine, who worked more than 6 hours online. Thus, the incidence of these symptoms was found to be positively associated with the duration of online activity.

### Mental health issues faced by students

Online education provided a medium to impart education during COVID-19 pandemic but it also isolated people, restricted them to a screen, and eliminated opportunities for real-time human interaction, resulting in a mental toll faced by the majority of the student population. Thus, students were asked in this study if they experienced any mental health issues during the COVID-19 period.

**Fig 4:**
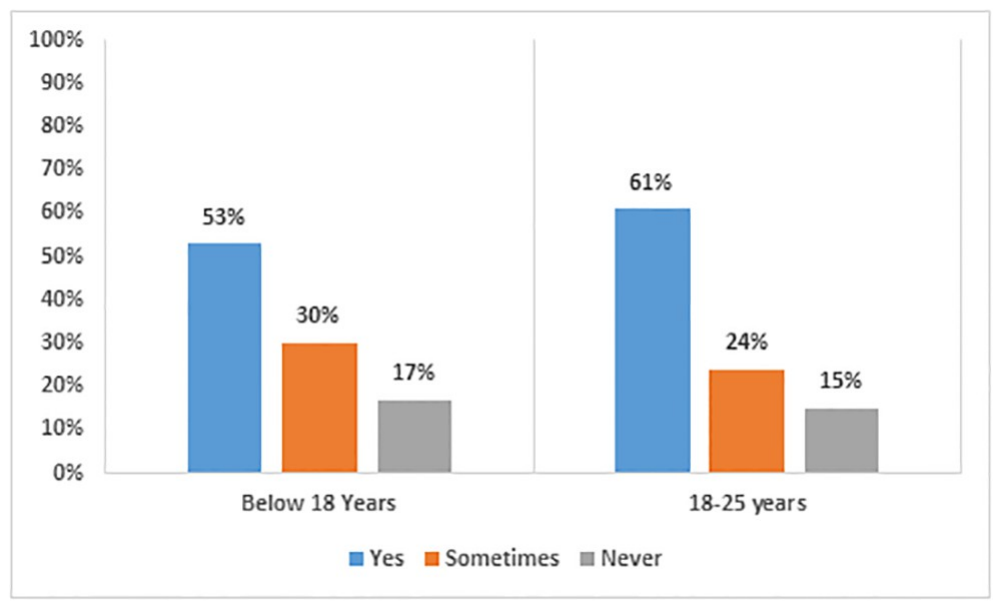
Mental health issues faced by students due to online learning.

Fig 4 indicates that 85.5% respondents aged 18 years and older primarily college-going students, reported experiencing mental health issues. Under the age of 18 years, that is, of school-aged students, approximately 83% faced mental health issues. The main reason behind this is reduced interaction with peers, teachers, and others. While everyone received uniform instructions and shared a similar educational environment, the familial environment and availability of resources at home varied for all of them, primarily in terms of resource accessibility and availability, parental non-financial support, and the home environment.

**Fig 5:**
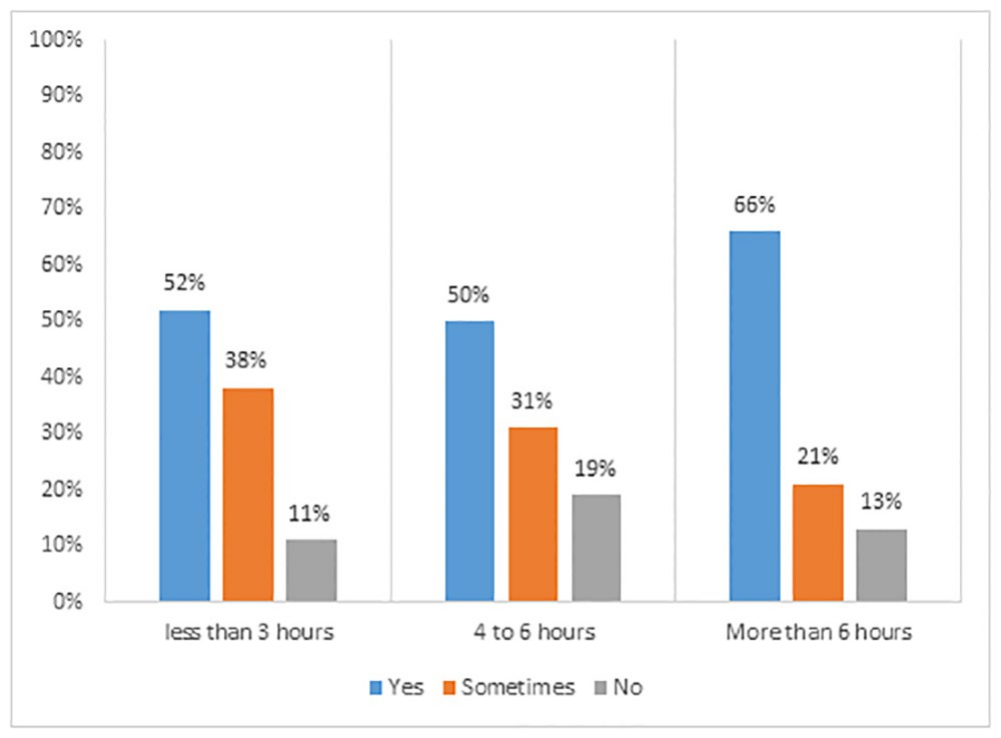
Number of working hours and frequency of reported mental health issue.

The study also tried to assess the relationship between the number of working hours and mental issues experienced by the students. Fig 5 indicates that 52% respondents who worked 3 hour or less online, faced some sort of mental issue while learning in the online sphere. This percentage increased positively to 66%, with the increasing number of hours spent online. Even after socioeconomic factors are taken into account, sedentary behavior has been shown to have a positive relationship with anxiety [40].

**Fig 6:**
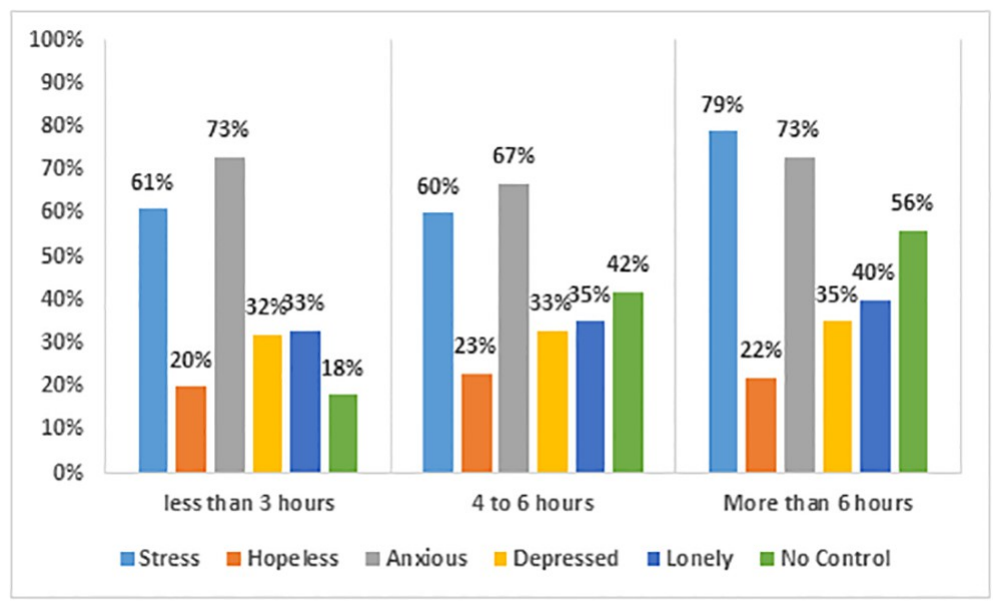
Types of mental health issues faced by students.

Fig 6 indicates the kinds of problems faced by students during online learning. Among students who spent more than 6 hours in an online learning environment, around 79% complained of being stressed and 73% of being anxious. Our observation was similar to a study conducted in Jordan where 69.5% respondents reported extreme psychological distress, while 54.9% reported no motivation to learn online [41].

COVID-19 pandemic affected, and has continued to impact the lives of students, immensely. It has become clear that students must possess specific survival skills, including problem-solving and critical thinking, to thrive in a stressful event such as the COVID-19 pandemic and to progress in their studies and prospective careers.

## Conclusion

Based on the aforementioned analysis of scholarly works and primary sources, it is evident that substantial deficiencies in the education domain, which were already there, have not only become more prominent but have also been intensified within the COVID-19 crisis. Looking ahead, students who have been left out of education must be targeted in future policies, as also the learning gap that has arisen as a result of insufficient knowledge imparted to students during the pandemic.

While some benefits are evident in premier institutions, with some students finding proactive opportunities in the online mode of function, the disproportionately higher number of negatively impacting situations is obvious. Most students were dissatisfied with the online mode of education and felt the need to use unconventional means to reach parental expectations or score high marks. Standards of invigilation also could not be maintained in the online mode, which made the use of unfair means easier for students.

Many students were opposed to continuing in the online mode after the pandemic because the transition from traditional methods to online was difficult for them. Students from premier institutions had the advantage of having previously worked with online teaching software, giving them an edge over other students, and thus the space to perform better and with ease. Students in premier institutions spent longer hours online due to other extracurricular and academic engagements, because they had access to better infrastructure, and longer possession of personal devices. Numerous reports have surfaced about online education being stressful and resulting in physical ailments. Over half the assessed demographic faced connectivity issues. The overall physical and mental health of students suffered greatly, with the majority of the demographic complaining of some kind of health issue they encountered during the excessive number of teaching hours they were required to spend online.

Educational institutions play a significant role in building resilient individuals and ascertaining that students have attained the requisite skill sets. The need of the hour is to develop solutions on a larger scale because delivering information is not the sole priority in times of crisis. Caring for and providing support is also critical for an individual’s well-being. Students are more likely to remember how they felt during these difficult moments rather than merely the instructional material supplied to them.

The prerequisite for a healthy learning environment is that families and teachers perform a crucial role in tackling challenges arising due to pandemics and in guiding children. The responsibility of parents is to provide their children with emotional support, while teachers need to act as mentors. These interventions can assist students by making the process of online learning more effective and valuable.

One of the essential policy focuses should be to keep students engaged within the learning process to limit unemployment. It is also imperative for governments and concerned stakeholders to be prepared to readily impart education to all students if a lockdown is imposed again in the future. In such times, it becomes pivotal to accommodate individual needs, support them with basic resources such as laptops and tablets, and provide a safer space for them to learn and thrive.

## Supporting information

S1 File (PDF)
