# Supplementary material for: Roadblocks in education amidst global crisis—A study based in India
Source: PLoS One. 2023 Oct 17;18(10):e0292465. doi: 10.1371/journal.pone.0292465 (PMC10581450; doi:10.1371/journal.pone.0292465)
Supplement: S1 File — (PDF) [file pone.0292465.s001.pdf]

Supplementary material

Online teaching fulfils the purpose			
**Response**	**Other Institutes**	**Premier Institutes**	**Grand Total**
Yes	26.45%	17.46%	21.96%
No	73.55%	82.54%	78.02%
**Grand Total**	**100.00%**	**100.00%**	**100.00%**

Did you appear for Online Assessment?			
**Response**	**Other Institutes**	**Premier Institutes**	**Grand Total**
Yes	86%	88%	87%
No	14%	12%	13%
**Grand Total**	**100%**	**100%**	**100%**

Satisfaction with online assessment			
**Response**	**Other Institutes**	**Premier Institutes**	**Grand Total**
Yes	30.63%	13.72%	22.48%
No	69.37%	86.28	77.52%
**Grand Total**	**100.00%**	**100.00%**	**100%**

Exposure to online learning before COVID-19			
**Response**	**Other Institutes**	**Premier Institutes**	**Grand Total**
Yes	12.53%	21.20%	16.76%
No	87.47%	78.81%	83.24%
**Grand Total**	**100.00%**	**100.00%**	**100%**

Willingness to use online learning after COVID-19			
**Response**	**Other Institutes**	**Premier Institutes**	**Grand Total**
Yes	14.85%	13.72%	14.30%
Can't Say	38.98%	39.65%	39.30%
No	46.17%	46.63%	46.39%
**Grand Total**	**100.00%**	**100.00%**	**100.00%**

Time spent on online learning			
**Time Spent in Hours**	**Other Institutes**	**Premier Institutes**	**Grand Total**
Less than 3	13.92%	1.00%	7.69%
4 to 6 hours	57.08%	44.39%	50.96%
More than 6 hours	29.00%	54.61%	41.35%
**Grand Total**	**100.00%**	**100.00%**	**100.00%**

Was online study hectic?			
**Response**	**Other Institutes**	**Premier Institutes**	**Grand Total**
Yes	52%	75%	63%
Sometimes	27%	17%	22%
No	21%	8%	15%
**Grand Total**	**100.00%**	**100.00%**	**100.00%**

Did you share Gadgets?			
**Response**	**Other Institutes**	**Premier Institutes**	**Grand Total**
Yes	65%	46%	56%
No	35%	53%	44%
**Grand Total**	**100.00%**	**100.00%**	**100.00%**

How was Internet connectivity?			
**Response**	**Other Institutes**	**Premier Institutes**	**Grand Total**
Very Good	54%	45%	50%
Good	14%	20%	33%
Poor	32%	34%	17%
**Grand Total**	**100.00%**	**100.00%**	**100.00%**

Did you attend online Webinars?			
**Response**	**Other Institutes**	**Premier Institutes**	**Grand Total**
Yes	59%	41%	72%
No	86%	14%	28%
**Grand Total**	**100.00%**	**100.00%**	**100.00%**

Were Webinars useful?			
**Response**	**Other Institutes**	**Premier Institutes**	**Grand Total**
Yes	56%	68%	62%
No	44%	32%	34%
**Grand Total**	**100.00%**	**100.00%**	**100.00%**

Physical health issue and number of online working hours				
**No. of working hours online**	**Faced physical health issue**			
	**Yes**	**Sometimes**	**No**	**Grand Total**
less than 3	46%	29%	25%	100%
4 to 6	57%	34%	9%	100%
More than 6	67%	31%	2%	100%
**Grand Total**	**57%**	**31%**	**12%**	**100%**

Type of physical health issue and number of online working hours			
Number of working hours online	Type of physical health issue		
	Giddiness, Migraine	Eye Strain	Back/ Neck pain
less than 3	31%	80%	45%
4 to 6	32%	75%	67%
More than 6	45%	92%	76%
**Grand Total**	**37%**	**82%**	**70%**

Mental health issue and number of online working hours				
Number of working hours online	Faced mental health issue			
	Yes	Sometimes	No	Grand Total
less than 3	52%	38%	11%	100%
4 to 6	50%	31%	19%	100%
More than 6	66%	21%	13%	100%
**Grand Total**	**57%**	**27%**	**16%**	**100%**

Type of Mental health issue and number of online working hours						
No. of working hours online	Type of mental health issue					
	Stress	Hopeless	Anxious	Depressed	Lonely	No Control
less than 3	61%	20%	73%	32%	33%	18%
4 to 6	60%	23%	67%	33%	35%	42%
More than 6	79%	22%	73%	35%	40%	56%
**Grand Total**	**68%**	**22%**	**71%**	**33%**	**36%**	**39%**
